# Altered expression of the voltage-gated calcium channel subunit α_2_δ-1: A comparison between two experimental models of epilepsy and a sensory nerve ligation model of neuropathic pain

**DOI:** 10.1016/j.neuroscience.2014.03.013

**Published:** 2014-12-26

**Authors:** M. Nieto-Rostro, G. Sandhu, C.S. Bauer, P. Jiruska, J.G.R. Jefferys, A.C. Dolphin

**Affiliations:** a Department of Neuroscience, Physiology and Pharmacology, University College London, London WC1E 6BT, UK; b Neuronal Networks Group, School of Clinical and Experimental Medicine, University of Birmingham, Birmingham B15 2TT, UK

**Keywords:** Ca_V_, voltage-gated calcium, DAPI, 4′,6-Diamidino-2-Phenylindole, DRG, dorsal root ganglion, EtOH, ethanol, HIV, human immunodeficiency virus, MAP2, microtubule-associated protein-2, PB, phosphate buffer, PBS, phosphate-buffered saline, PSNL, partial sciatic nerve ligation, SNL, spinal nerve ligation, calcium channel, dorsal root ganglion (DRG), alpha2delta subunit, epilepsy, neuropathic pain, reactive gliosis

## Abstract

The auxiliary α_2_δ-1 subunit of voltage-gated calcium channels is up-regulated in dorsal root ganglion neurons following peripheral somatosensory nerve damage, in several animal models of neuropathic pain. The α_2_δ-1 protein has a mainly presynaptic localization, where it is associated with the calcium channels involved in neurotransmitter release. Relevant to the present study, α_2_δ-1 has been shown to be the therapeutic target of the gabapentinoid drugs in their alleviation of neuropathic pain. These drugs are also used in the treatment of certain epilepsies. In this study we therefore examined whether the level or distribution of α_2_δ-1 was altered in the hippocampus following experimental induction of epileptic seizures in rats, using both the kainic acid model of human temporal lobe epilepsy, in which status epilepticus is induced, and the tetanus toxin model in which status epilepticus is not involved. The main finding of this study is that we did not identify somatic overexpression of α_2_δ-1 in hippocampal neurons in either of the epilepsy models, unlike the upregulation of α_2_δ-1 that occurs following peripheral nerve damage to both somatosensory and motor neurons. However, we did observe local reorganization of α_2_δ-1 immunostaining in the hippocampus only in the kainic acid model, where it was associated with areas of neuronal cell loss, as indicated by absence of NeuN immunostaining, dendritic loss, as identified by areas where microtubule-associated protein-2 immunostaining was missing, and reactive gliosis, determined by regions of strong OX42 staining.

## Highlights

• The calcium channel subunit α_2_δ-1 binds the antiepileptic drug gabapentin.
• We examined if α_2_δ-1 was altered in two animal models of temporal lobe epilepsy.
• In the kainate model there was local reorganization of α_2_δ-1 immunostaining.
• This was associated with areas of hippocampal neuronal loss and reactive gliosis.
• Unlike in neuropathic pain models, α_2_δ-1 did not increase in these epilepsy models.

## Introduction

Voltage-gated calcium (Ca_V_) channels consist of three subgroups, the Ca_V_1, 2 and 3 classes (Catterall, 2011). Most of these channels, apart from Ca_V_1.1 which is a skeletal muscle channel, are involved in neuronal function, with their most prevalent functions being in excitation–transcription coupling (Ca_V_1.2), synaptic transmission (Ca_V_2 channels), and regulation of neuronal excitability and pacemaker activity (Ca_V_3 channels). Because of their key roles in neuronal function, it is not surprising that a number of different calcium channels have been implicated in the pathogenesis of various forms of epilepsy, in both humans and in animal models. These channels include T-type (Ca_V_3) channels (Su et al., 2002, Tringham et al., 2012, Cheong and Shin, 2013), P/Q-type (Ca_V_2.1) channels (Imbrici et al., 2004, Rajakulendran et al., 2012), and the auxiliary subunits, β4 (Escayg et al., 1998) and α_2_δ-2 (Barclay et al., 2001, Edvardson et al., 2013). Furthermore several calcium channels are either actual or potential targets for therapeutic intervention (Cain and Snutch, 2012, Powell et al., 2013).

The Ca_V_ auxiliary α_2_δ and β subunits, both of which have four isoforms, are associated with the “high voltage activated” Ca_V_1 (L-type) and Ca_V_2 classes (N, P/Q and R-type) of calcium channel, but are not thought to be associated with Ca_V_3 (T-type) calcium channels. Both auxiliary subunits increase plasma membrane expression of the Ca_V_1 and Ca_V_2 channels, and influence their biophysical properties (Dolphin, 2012b). Of relevance to the potential pathological roles of α_2_δ subunits, the α_2_δ-1 isoform is up-regulated following peripheral somatosensory nerve damage (for review see Bauer et al., 2010), whereas mutations in α_2_δ-2 have been linked to absence epilepsy (Barclay et al., 2001, Dolphin, 2012a, Edvardson et al., 2013). The α_2_δ proteins have also been reported to fulfill other functions independent of calcium channels (Eroglu et al., 2009, Kurshan et al., 2009), and are likely to interact with other binding partners, including thrombospondins (Eroglu et al., 2009).

Both α_2_δ-1 and α_2_δ-2 represent binding sites for the anti-epileptic α_2_δ ligand drugs gabapentin and pregabalin (Brown et al., 1998, Klugbauer et al., 2003). These drugs are used as adjunct therapy in several forms of epilepsy, particularly drug-resistant partial seizures (Marson et al., 2000, Arroyo et al., 2004, Taylor et al., 2007). They are also widely used in the treatment of neuropathic pain resulting from peripheral nerve damage of various origins, such as trauma, trigeminal neuralgia, diabetes-induced nerve damage, and chronic pain following viral infection, including post-herpetic neuralgia (Moore et al., 2009, Moore et al., 2011). They have also been used for alleviation of chronic pain resulting both from human immunodeficiency virus (HIV) infection and as a side effect of some of the anti-HIV drugs (Schutz and Robinson-Papp, 2013). Chronic neuropathic pain resulting from cancer chemotherapeutic drugs, including paclitaxel and cisplatin, is also treated with gabapentinoid drugs (Fallon, 2013).

The mechanism of action of the gabapentinoid drugs in the treatment of epilepsies remains unclear. In this study we wished to examine whether the level or distribution of α_2_δ-1 was altered following experimental induction of epileptic seizures in rats, since a change in α_2_δ-1 level or distribution might contribute to the anti-epileptic mechanism of action of gabapentinoid drugs, in a similar way to their therapeutic action in neuropathic pain (see Discussion). It was not possible to examine changes in distribution of α_2_δ-2 protein in parallel in this study, as no antibodies suitable for immunohistochemistry are currently available.

We chose to use the rat kainic acid model of human temporal lobe epilepsy, in which spontaneous seizures have been found to occur following a latent period after the initial induction by kainic acid of persistent seizures, known as status epilepticus (Buckmaster and Dudek, 1997). In this model, rats first develop status epilepticus, and then consistently develop spontaneous seizures which exhibit a gradual increase in spontaneous frequency in the subsequent weeks (Dudek and Staley, 2012). This model is relevant because gabapentin is known to be effective against seizures induced by this means (Cilio et al., 2001). For comparison, we also used the tetanus toxin model of temporal lobe epilepsy (Jefferys et al., 1995), in which status epilepticus is not induced (Finnerty and Jefferys, 2002).

## Experimental procedures

### Kainic acid treatment

Ten adult male Sprague–Dawley rats weighing approximately 250 g were injected with kainic acid (2.5 mg/kg i.p.) to induce status epilepticus. Injections were repeated once per hour until 5–9 Racine stage III/IV/V seizures per hour have occurred (Luttjohann et al., 2009). After 40–60 min from the onset of status epilepticus (near-continuous motor epileptic activity), diazepam (10 mg/kg, i.p.) was injected repeatedly until continuous motor activity disappeared. Following status epilepticus animals were housed separately. When seizures stopped, diazepam (2.5 mg/kg, i.p.) was continued every 30 min. Subcutaneous administration of warmed sterile saline was given if the animals appeared lethargic and/or a significant drop in weight occurred. Rats were housed in single cages under standard conditions in a room with controlled temperature (22 ± 1 °C) and 12/12-h light/dark cycle. The animals had *ad libitum* access to food and water. Immediately following the status epilepticus, rats were manually fed, if necessary until adequate recovery, and provided with standard food and also mashed food and apple slices. Control animals were treated with an equivalent volume and number of injections of sterile saline.

### Unilateral intrahippocampal injection of tetanus toxin

Four rats were injected with tetanus toxin and four rats with saline as controls. Surgical preparation was performed as previously described (Jiruska et al., 2013), under ketamine/xylazine anesthesia. A small trephine opening was drilled over the right hippocampus at coordinates 4.1 mm caudal to bregma and 3.9 mm laterally (Paxinos and Watson, 2005). Using a Hamilton microsyringe and infusion pump (KD Scientific Inc., Holliston, USA) 1 μl of tetanus toxin (Sigma–Aldrich, Poole, UK) solution was injected into the stratum radiatum of the right hippocampal CA3 area (depth 3.9 mm). The tetanus toxin solution contained 25 ng of tetanus toxin in 1 μl of 0.05 M phosphate-buffered saline (PBS; Sigma–Aldrich, UK) and 2% bovine serum albumin (Sigma–Aldrich, UK). It was injected at 200 nl/min. The microsyringe was left in the hippocampus for 5 min after the injection ended to avoid the solution leaking back through the injection track. Control animals were injected with 1 μl of 0.05 M PBS with 2% bovine serum albumin. Following surgery, the rats were housed in single cages and allowed to recover for 2 days. Subsequently they were monitored for spontaneous seizures in video monitoring units to verify the development of spontaneous and recurrent seizures. Videos were recorded using digital infra-red cameras (Y-cam Solutions Ltd., Richmond, UK). Animals were video-monitored for 4 weeks.

All animal procedures were licensed and performed in strict accordance with the Animal Scientific Procedures Act (1986) of the United Kingdom and with Birmingham University Ethical Review.

### Sample preparation and immunohistochemistry

Rats were deeply anesthetized with an intraperitoneal injection of (600 mg/kg) pentobarbitone (Euthatal, Merial Animal Health, Harlow, UK), perfused transcardially with saline containing heparin, followed by perfusion with 4% paraformaldehyde in 0.1 M phosphate buffer (PB, pH 7.4). Brains were dissected and the tissue was post-fixed for 1.5–2 h, washed with PB, cryoprotected by incubation in PB with 15% sucrose, and finally frozen before embedding in optical cutting temperature compound (OCT) and sectioning with a cryostat. Serial coronal sections of 25 μm of the brain region including the hippocampus were collected and placed sequentially on a series of six slides, with 4 sections/slide; the distance between each section and the next on any slide was therefore 150 μm. A total of at least eight such series were collected per animal.

For Cresyl Violet staining, the first slide of each series was consecutively immersed for 5 min in PBS, 50%, and 75% ethanol (EtOH) and then stained in 0.1% Cresyl Violet (Sigma) for 15 min; after washing in H_2_O, the slides were briefly immersed in 75% EtOH, 0.3% acetic acid, dehydrated, cleared in Histoclear for 5 min and mounted in DPX, neutral mounting medium (Sigma–Aldrich).

For immunofluorescence labeling to detect α_2_δ-1, sections underwent heat-induced antigen retrieval (10 mM citrate buffer, pH 6.0, 0.05% Tween 20, 98 °C for 10 min) prior to blocking with 10% goat serum in the presence of 0.1% Triton X-100 in PBS for 1 h. Sections were then incubated with the mouse monoclonal anti-α_2_-1 antibody (Sigma, 1:100) in 50% blocking buffer for 2 or 3 days at 4 °C. These sections were also stained for microtubule-associated protein-2 (chicken Ab against MAP2, EnCor Biotechnology, Gainesville, FL, 1:1000). After extensive washing with PBS containing 0.1% Triton X-100, sections were incubated with biotinylated goat anti-mouse IgG (1:500) overnight at 4 °C and Streptavidin–Alexa Fluor 488 (1:500) and goat anti-chicken ab coupled to Alexa Fluor 647 (1:500) overnight at 4 °C. Samples processed for NeuN immunoreactivity (mouse monoclonal NeuN Ab, Millipore, 1:500) and OX42 immunoreactivity (mouse monoclonal OX42 Ab, Abcam, 1:200) were treated in the same way except that they did not require antigen retrieval. Samples were then washed and all were stained with 4′,6-Diamidino-2-Phenylindole (DAPI), before mounting in VectaShield (Vector Laboratories, Burlingame, CA, USA).

In the kainic acid study, experiments were performed on a total of 16 rats (10 kainate-treated and six saline-treated, Table 1), of which all were analyzed for histological abnormalities in the hippocampus by staining the first slide of each series with Cresyl Violet. This staining was therefore performed on at least 8 slides per animal. Slides from 10 kainate-treated rats and four saline-treated were stained for α_2_δ-1 and MAP2 immunoreactivity, selecting one slide from each of 2–3 series adjacent to those showing neuronal cell disruption from the Cresyl Violet staining. Adjacent slides were then processed for NeuN and OX42 staining (one slide each from one representative series per animal; from six kainate-treated and three saline-treated rats). In the tetanus toxin study, four tetanus toxin-injected and four saline-injected rats were processed similarly (Table 2).

**Table 1:** Kainic acid study: summary of animals used and histological analysis

ID	Treatment	Status Epilepticus	Hippocampal damage (Cresyl Violet)	Microglia activation (OX42)	Hippocampal neuronal cell loss (NeuN)	Disruption of α_2_δ-1 staining in areas of neuronal cell loss	Ectopic α_2_δ-1 staining in CA3
1	Kainic acid	Yes	CA1 (bi) + CA3 (bi)	ND	ND	No	No
2	Kainic acid	Yes	CA1 (bi) + CA3 (bi)	Yes	Yes	Yes (CA1)	No
3	Kainic acid	Yes	CA3 (bi)	ND	ND	No	No
4	Kainic acid	No	CA1 (bi) + CA3 (bi)	Yes	Yes	No	++
5	Kainic acid	Yes	CA1 + CA3 (bi)	ND	ND	No	No
9⁎	Kainic acid	Yes	CA1 + CA3 (bi)	Yes	Yes	Yes (CA1)	++
10	Kainic acid	Yes	CA3 (bi)	ND	ND	No	No
11	Kainic acid	Yes	CA1 + CA3 (bi)	Yes	Yes	Yes (CA1)	No
12	Kainic acid	No	No	No	No	No	No
13	Kainic acid	Yes	CA1 (bi) + CA3 (bi)	Yes	Yes	Yes (CA1)	+

6	Saline	No	No	ND	ND	No	No
7	Saline	No	No	No	No	No	No
8	Saline	No	No	ND	ND	ND	ND
14⁎⁎	Saline	No	No	ND	ND	ND	ND
15	Saline	No	No	No	No	No	++
16	Saline	No	No	No	No	No	No

Summary of all rats used in the kainic acid study, their treatment and histological analysis. Cresyl Violet staining was performed in the first slide of each series (equivalent to at least 8 slides per animal) for all brains. The presence of neuronal cell death was detected in the pyramidal cell layers of CA1 and/or CA3, as stated. In most cases the damage appeared bilaterally (bi). In the kainic acid-treated rats, sections adjacent to those Cresyl Violet stained sections showing hippocampal damage were also stained for α_2_δ-1, OX42 and NeuN, in a subset of rats. ND = not determined. +: staining present; ++: strong staining.

⁎ Sample with rostral side of right hippocampus deformed.

⁎⁎ Sample not well-perfused.

**Table 2:** Tetanus toxin study: summary of animals used and histological analysis

ID	Treatment	Status epilepticus	Spontaneous seizures	Hippocampal damage near injection site (DAPI)	Microglia activation near injection site (OX42)	Microglia activation contralateral to injection site (OX42)	Disruption of α_2_δ-1 staining in areas of neuronal damage
TTX4	Tetanus toxin	No	Yes (early onset)	DG + CA1	CA1	No	No
TTX5	Tetanus toxin	No	Yes (infrequent)	CA1	CA1	No	CA1
TTX7	Tetanus toxin	No	Yes (late onset)	CA1	CA1	+	CA1
TTX8	Tetanus toxin	No	No	DG	CA1 + DG	No	No

Sal1	Saline	No	No	CA1 + DG	CA1	No	No
Sal2	Saline	No	No	CA1	CA1 + DG	No	CA1
Sal3	Saline	No	No	CA1 + DG	CA1 + DG	No	No
Sal4	Saline	No	No	CA1	No	No	No

Summary of rats used in the tetanus toxin study, their treatment and histological analysis.

### Image acquisition and analysis and composite assembly

Cresyl Violet staining was visualized on a Leica MZ7.5 stereomicroscope with a DC300 camera under transmitted light using the Leica IM50 software (Leica, Milton Keynes, UK). To produce the hippocampus composites immunofluorescence images were acquired with Volocity using an inverted fluorescence microscope Axiovert 500 M with a 5× objective, converted to JPEGs and mounted using Autostich software (www.autostich.net), or manually if the level of signal was too low for the software. For higher resolution, confocal images were acquired in a Zeiss LSM 500 M with a 10× objective (15 μm optical sections) using Zeiss LSM Image acquisition software.

### Spinal nerve ligation (SNL) experiments

These data were obtained during the course of a previous study (Bauer et al., 2009), but the images in Fig. 6 were not included in that study. All the experimental detail is identical to that previously described (Bauer et al., 2009).

## Results

### Gross morphology and cell loss in the hippocampus following kainic acid treatment

Rats were repeatedly injected with kainic acid (2.5 mg/kg, i.p., *n* = 10), until status epilepticus was observed, which occurred in 8/10 kainic acid-treated rats (Table 1). For the control rats, saline was administered (*n* = 6; Table 1). Rats were then perfused and the brains removed after 5 weeks.

The gross morphology of all the hippocampi was examined with Cresyl Violet staining, in order to determine whether neuronal cell death had occurred. Immunostaining was then performed for α_2_δ-1 and the dendritic marker MAP2 in all 10 kainic acid-treated and four of the saline-treated rats. In six kainate-treated and three saline-treated rats, immunostaining was also performed for the microglial marker OX-42 (Shaw et al., 1990) and the neuronal nuclear marker NeuN.

**Fig. 1:**
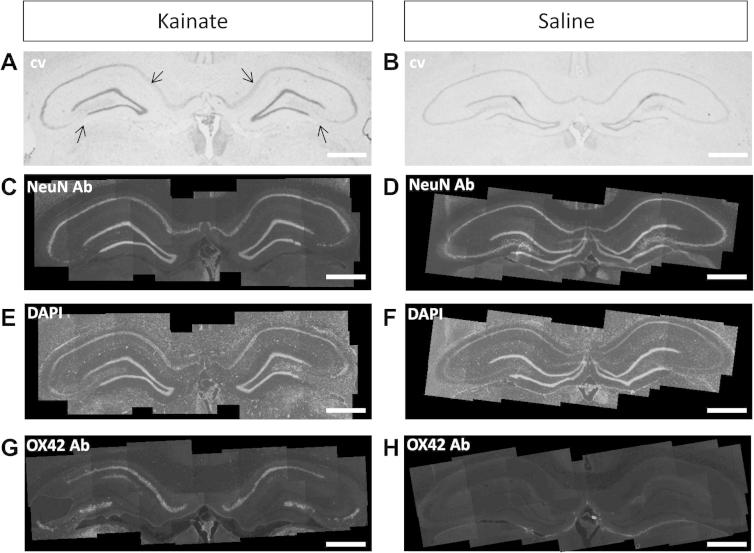
Comparison between hippocampi from kainic acid-treated and control rats. (A, B) Cresyl Violet (cv) staining of a representative hippocampal section from a kainate-treated rat (A, rat #2) and a saline control rat (B, rat #16). Arrows indicate cell loss in A. (C, D) Composite image of NeuN immunostaining of a representative hippocampal section from a kainate-treated rat (C, rat #2) and a saline control rat (D, rat #16). (E, F) Composite image of DAPI staining of a representative hippocampal section from a kainate-treated rat (E, rat #2) and a saline control rat (F, rat #16). (G, H) Composite image of OX42 staining in adjacent hippocampal sections from kainate-treated rat (G, rat #2) or saline control rat (H, #16). Scale bar = 1 mm for all panels.

We were interested in whether kainate-induced seizures and neuronal damage were associated with an altered expression of α_2_δ-1. From an examination of gross morphology using Cresyl Violet staining, we found regions of neuronal cell loss in CA1, CA3 and the hilus of the dentate gyrus in 8/8 kainate-treated animals which had developed status epilepticus (Fig. 1A, arrows; Table 1), and 1/2 kainate-treated rats which did not develop status epilepticus (Table 1), but not in any saline-treated rats (Fig. 1B and Table 1). This neuronal cell loss was confirmed by comparing NeuN immunostaining, which stains neuronal nuclei (Fig. 1C, D), with DAPI staining (Fig. 1E, F), which stains all nuclei.

### Expression of OX42 in hippocampus following kainic acid treatment

We found that expression of OX42, a marker of microglial activation (Robinson et al., 1986), was present in the hippocampus of 4/4 kainate-treated rats examined that had experienced status epilepticus (Fig. 1G) and in 1/2 rats that did not develop status epilepticus. Expression of OX42 was seen in both CA1 and CA3 regions (Fig. 1G), as well as elsewhere in the brain (data not shown). The location of neuronal damage, determined by loss of NeuN staining was generally associated with the appearance of OX42 staining (compare Fig. 1C, G). In contrast, no OX42 staining was observed in 3/3 hippocampi examined of saline-treated controls or 1/2 kainate-treated rat that did not develop status epilepticus and did not show any brain damage (Table 1 and Fig. 1H).

### Expression of α_2_δ-1 in hippocampus following kainic acid treatment

**Fig. 2:**
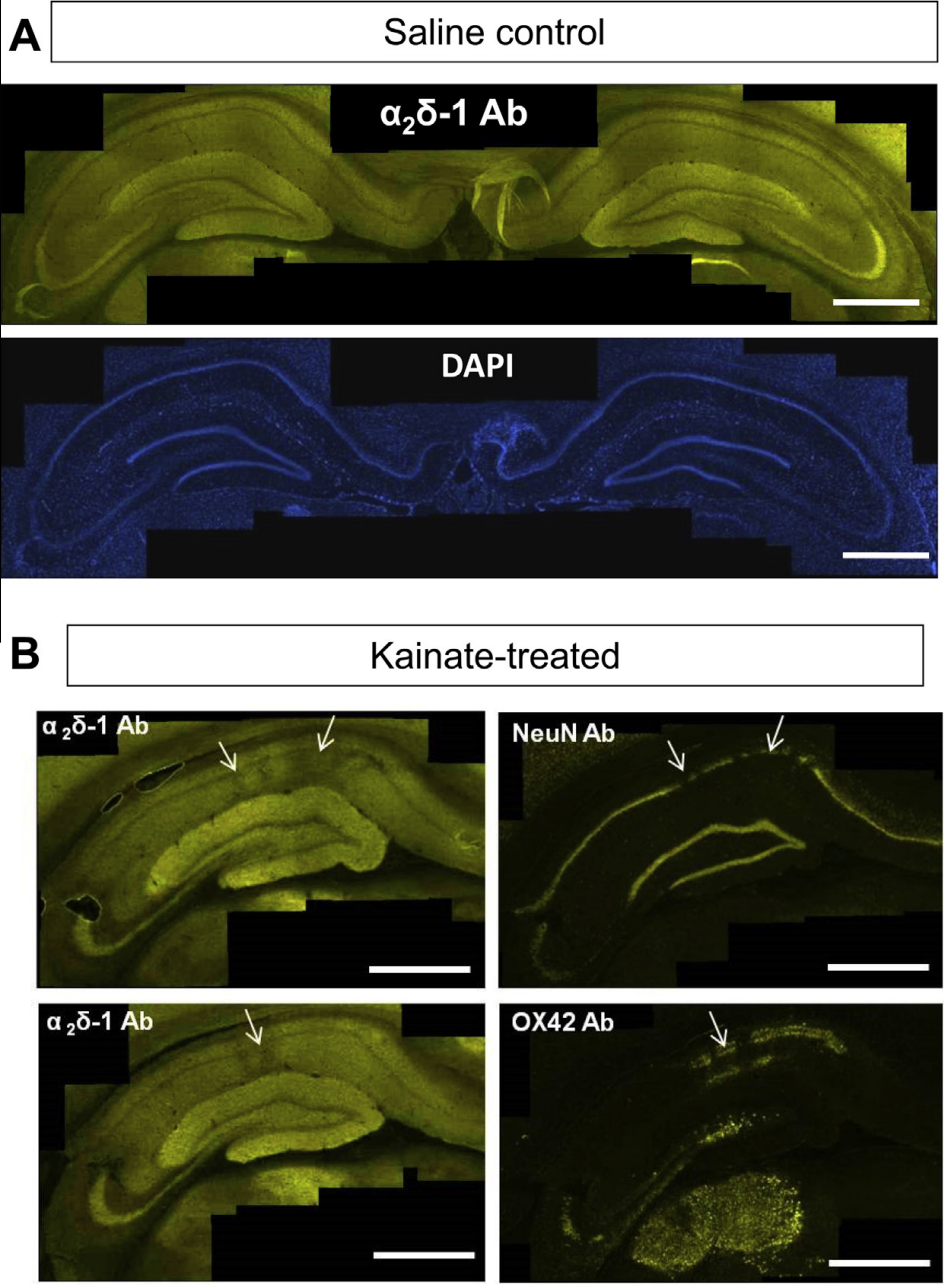
α_2_δ-1 immunostaining in kainic acid-treated rat hippocampus compared to control hippocampus. (A) Low power composite image of α_2_δ-1 immunostaining (upper panel) in saline control (rat #6), compared with DAPI staining (lower panel). Scale bars = 1 mm. (B) Low power composite image of staining in hippocampus of a kainate-treated rat (#11) for α_2_δ-1 (left, upper and lower panels are 150 μm apart), NeuN (right upper panel) and OX42 (right lower panel), showing patchy loss of α_2_δ-1 immunoreactivity, associated with regions of loss of CA1 pyramidal neurons, and up-regulation of OX42 immunoreactivity (arrows). The sections stained for NeuN and OX42 are consecutive to the upper left section stained for α_2_δ-1. Scale bars = 1 mm.

The α_2_δ-1 subunit has been identified previously as a synaptic protein, present particularly in presynaptic terminals (Taylor and Garrido, 2008, Bauer et al., 2009). However, α_2_δ-1 is also associated with calcium channels, including L-type calcium channels, present on dendrites (Schlick et al., 2010). We found α_2_δ-1 to be expressed throughout the hippocampus (Fig. 2) although largely absent from the main cell body layers, including the granule cells of the dentate gyrus, and the CA3 and CA1 pyramidal cell layers. The α_2_δ-1 protein is particularly strongly expressed in the molecular layer and hilus of the dentate gyrus and in the CA3 stratum lucidum, but also in the stratum oriens and stratum radiatum of the CA1 region (Fig. 2A). This distribution is similar to that found previously using paraffin-embedded rat brain sections and Horseradish Peroxidase staining (Taylor and Garrido, 2008).

Since α_2_δ-1 is elevated following peripheral nerve damage in both sensory neurons (Luo et al., 2001, Newton et al., 2001, Bauer et al., 2009) and in motor neurons (Bauer et al., 2009; see also Fig. 6), we examined whether α_2_δ-1 expression was altered in the hippocampus of kainate-treated rats. We did not observe any consistent increase of α2δ-1 staining in any region of the hippocampus of any of the kainate-treated rats; on the contrary there was localized loss of α_2_δ-1 immunostaining in the CA1 region, occurring in patches (Fig. 2B, left panels, arrows), at sites correlating with CA1 pyramidal neuronal cell loss, shown by NeuN staining (Fig. 2B, upper right panel, arrow). These patches are also associated with regions of reactive gliosis, indicated by OX42 staining (Fig. 2B, lower right panel, arrow).

**Fig. 3:**
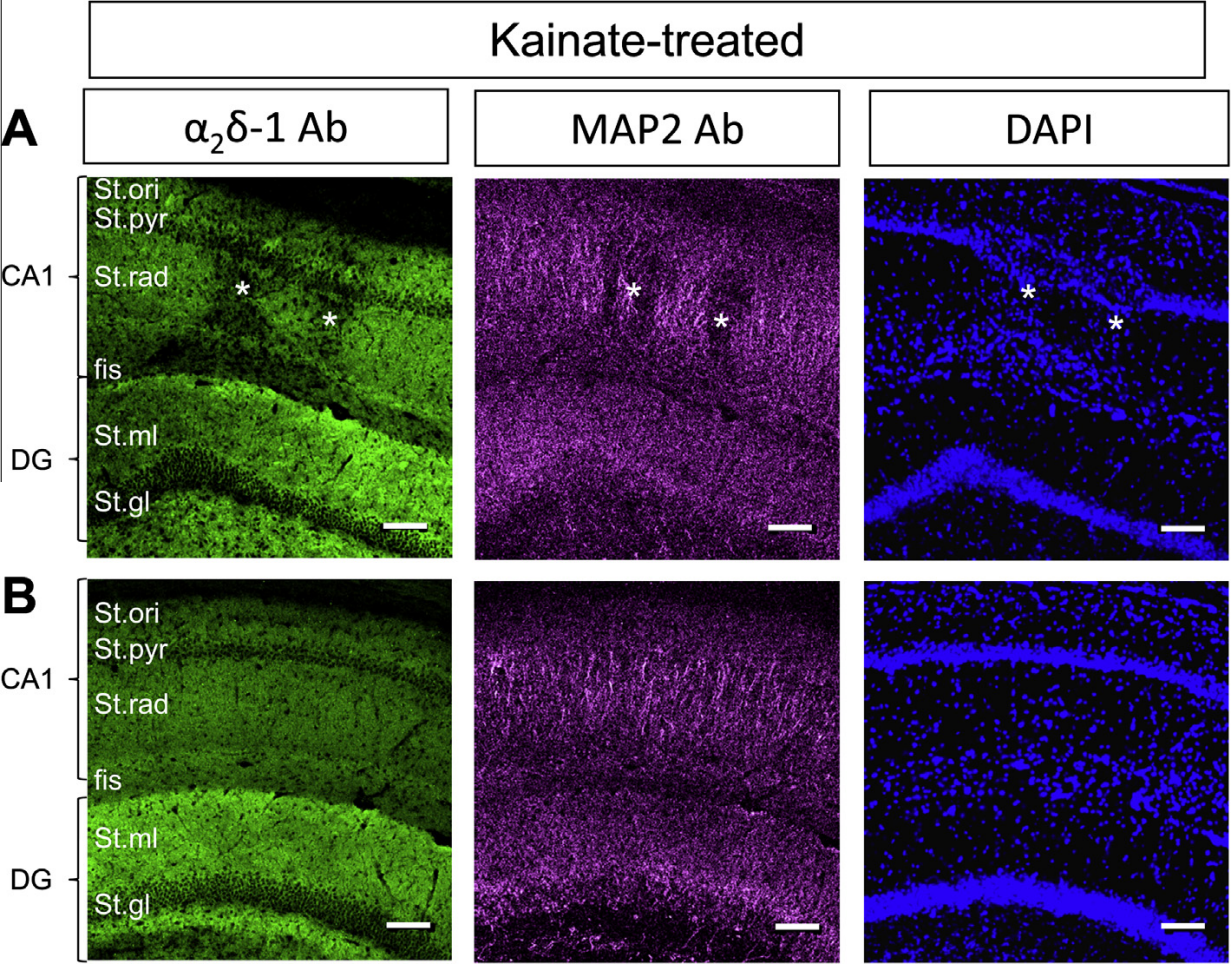
Localized reduction of α_2_δ-1 in stratum radiatum CA1 in field associated with loss of pyramidal neurons in kainic acid-treated rats. (A, B) High power images of α_2_δ-1 (left panel), MAP2 (middle panel), DAPI (right panel) in sections of CA1 and dentate gyrus (DG) associated with neuronal loss. (A) Shows reduced α_2_δ-1 immunoreactivity in localized areas (asterisks) where dentritic branches are absent, shown by loss of MAP2 immunoreactivity, and DAPI staining shows glial proliferation around the site of pyramidal neuron death. (B) Shows a section 1.2 mm rostral to the damaged area, as a control. *Abbreviations:* St.Ori = stratum oriens, St.pyr = stratum pyramidale, St.rad = stratum radiatum, fis = hippocampal fissure, St.ml = molecular layer, St.gl = granule cell layer. Scale bars = 100 μm.

At higher power magnification (Fig. 3), the patches associated with loss of α_2_δ-1 immunoreactivity are also associated with a reduction in MAP-2 staining, indicative of a loss of pyramidal cell dendrites, and an increase in DAPI staining, likely to be associated with reactive gliosis (starred areas in Fig. 3A). In Fig. 3B, a region from the same hippocampus, 1.2 mm rostral to the damaged area, is shown as a control.

**Fig. 4:**
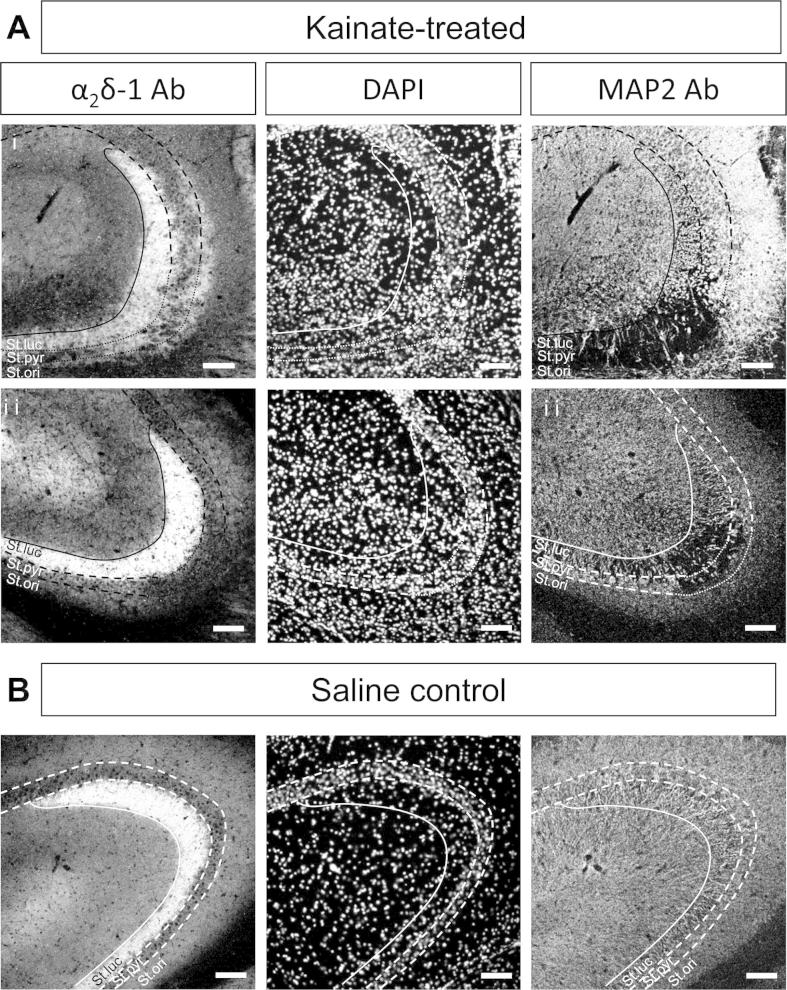
α_2_δ-1 staining in the stratum lucidum remains unaffected by neuronal cell loss in CA3 stratum pyramidale. (A, B) High power images of α_2_δ-1 (left panel), DAPI (middle panel), and MAP2 (right panel), in two example sections of the CA3 region of hippocampus from two kainate-treated rats (Ai, #4 and Aii, #13) and a saline-treated rat (B, #6). For clarity the stratum lucidum is delimited by a solid line and the stratum pyramidale by a dashed line, or dotted line in areas of neuronal cell loss. *Abbreviations:* St.luc = stratum lucidum, St.pyr = stratum pyramidale, St.ori = stratum oriens. Scale bar = 100 μm.

Despite extensive pyramidal neuron loss from the CA3 region, in the tissue from 9/10 kainic acid-treated rats (Figs. 1A, C, E, 2B, 3A and 4A), we found that the strong α_2_δ-1 immunostaining in the CA3 stratum lucidum was undiminished in these hippocampi (Fig. 4). In Fig. 4Ai and ii, the CA3 regions from two kainate-treated rats are shown in comparison with the same region from a saline-treated rat (Fig. 4B). The CA3 stratum lucidum showing intense α_2_δ-1 staining corresponds to the layer containing mossy fiber terminal synapses. In some sections, expression of α_2_δ-1 was also observed to be associated with the stratum oriens of the CA3 region in kainate-treated rats (Fig. 4Ai). This was observed in 3/10 kainate-treated rats (Table 1), but we also observed strong α_2_δ-1 staining in the stratum oriens of one of the saline-treated rats (Table 1).

### Effect of intrahippocampal tetanus toxin on OX42 and α_2_δ-1 staining

For comparison with the kainic acid model, we examined four rats given an intra-hippocampal injection of tetanus toxin, with four rats given saline injection as controls. Three of the tetanus toxin-treated animals developed spontaneous seizures (Table 1). None of the saline-treated rats developed seizures.

**Fig. 5:**
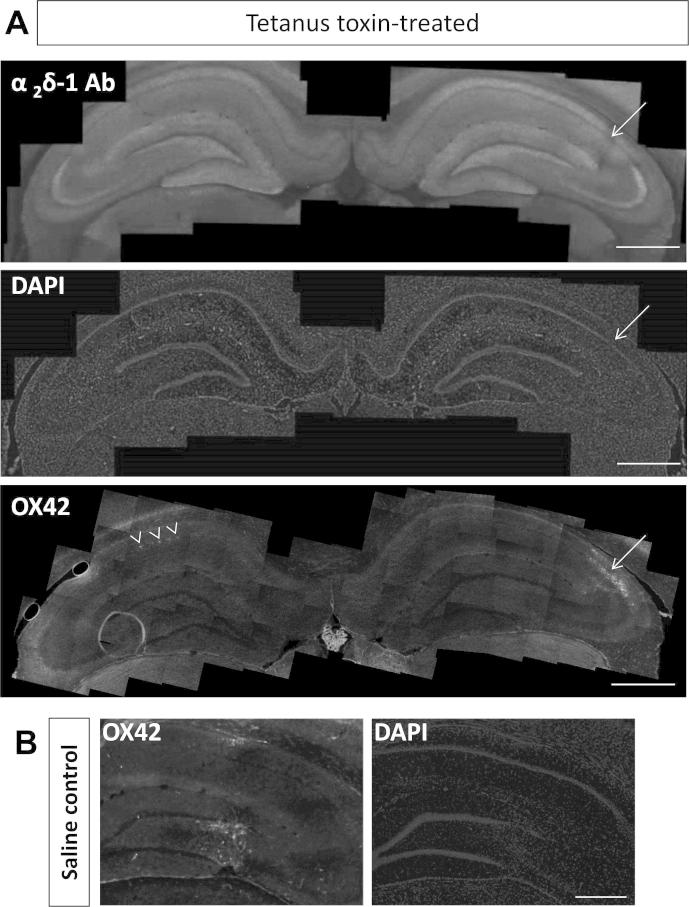
Histological examination of tetanus toxin-injected rat hippocampus, compared to saline-injected control. (A) Low power composite images in the hippocampus of a tetanus toxin-treated rat (TTX7), of α_2_δ-1 immunostaining (top panel) showing a localized area of reduced α_2_δ-1 staining (arrow), DAPI staining (middle panel, same section as top panel) showing pyramidal cell layer disruption due to injection track (arrow), and OX42 staining (bottom panel) from a section 175 μm away from that shown in upper and middle panels, showing microglial activation around injection site (arrow) and also a small amount along the stratum pyramidale of CA1 of the non-injected side (arrowheads). Scale bars = 1 mm. (B) Low power composite images of OX42 immunostaining and DAPI staining in the hippocampus (left and right panels, respectively) around the injection track of a saline-injected rat (Sal3). Scale bars = 0.5 mm.

Hippocampal sections from all rats were subjected to immunohistochemical analysis of α_2_δ-1 and OX42 immunoreactivity, and counterstained with DAPI. The pattern of expression of α_2_δ-1 did not show any significant change in any of the samples, except a localized reduction of immunoreactivity in CA1, coinciding with the injection track in two of the tetanus toxin-treated rats (Fig. 5A, top and middle panels, arrowed), and one saline-treated rat (data not shown). OX42 signal also appeared along the injection track in all the tetanus toxin-treated (Fig. 5A, bottom panel, arrow) and 3/4 saline-treated rats (Fig. 5B left panel). A minor contralateral expression of OX42 in CA1 stratum pyramidale was observed in one of the four tetanus toxin-treated rats that developed late seizures (Fig. 5A, bottom panel, arrowheads). DAPI staining revealed that in all cases, there was some cell loss in the CA1 stratum pyramidale and/or lateral dentate gyrus, associated with the injection track (Fig. 5A middle panel and 5B right panel), and this neuronal cell death coincided with the areas showing OX42 immunoreactivity and reduced α_2_δ-1.

### Up-regulation of α_2_δ-1 following peripheral nerve injury

**Fig. 6:**
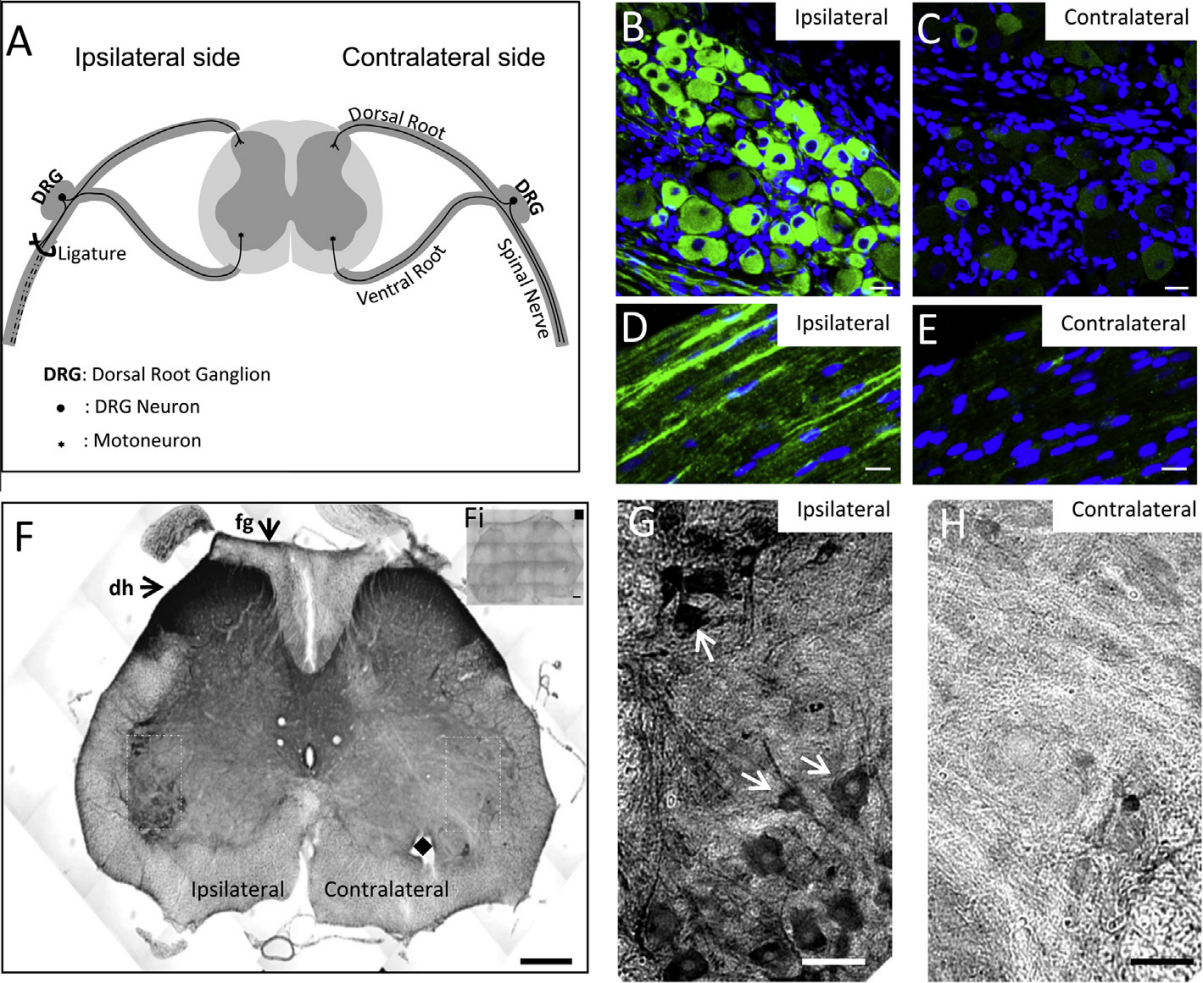
α_2_δ-1 up-regulation following SNL in rat DRGs and their projections, and in motor neurons. (A) Diagram of the SNL model used to induce mechanical hyperalgesia. Ipsilateral ligation (ligature, left) of the spinal nerve at the level of L5 (and L6, not shown) affects DRG neurons and their afferent projections innervating the dorsal horn of the spinal cord. Black circles indicate the location of DRG cell bodies, small black stars indicate motor neuron cell bodies. (B–E) Representative α_2_δ-1 immunofluorescence images of L6 DRG section (B and C, ipsi- and contralateral respectively) and dorsal root section (D and E, ipsi- and contralateral respectively), 14 days after SNL. α_2_δ-1 is upregulated in the ipsilateral DRG neurons of all sizes and also in their projections (green: α_2_δ-1, blue: DAPI). Scale bars = 20 μm. (F) Montage of α_2_δ-1 immunoperoxidase-stained microphotographs covering a complete L5 spinal cord section 14 days after SNL. α_2_δ-1 immunoreactivity is increased ipsilaterally, in the dorsal horn (dh), the fasciculus gracilis (fg), and in the cell bodies of motor neurons of the ventral side compared to the contralateral side. White dashed boxes indicate areas shown in G and H. Diamond (♦) marks a hole in the tissue introduced to identify the contralateral site. Scale bar = 200 μm. Fi: Insert shows the absence of staining when the primary antibody was omitted. (G, H) Higher power images of the ventral spinal cord, ipsilateral (G) and contralateral (H) to SNL, showing increased α_2_δ-1 immunostaining in motor neurons on the ipsilateral side (e.g. white arrows in G), Scale bars = 50 μm. The data for the figure were obtained during the course of our previous study (Bauer et al., 2009), but the images were not included in that study.

In contrast to the results described above, following physical insult to peripheral axons of somatosensory nerves, such as SNL (Fig. 6A) and partial sciatic nerve ligation (PSNL), α_2_δ-1 mRNA and protein is rapidly and strongly up-regulated on the injured side in dorsal root ganglion (DRG) neuron somata (Fig. 6B compared to 6C), axons (Fig. 6D compared to 6E) and terminals (Fig. 6F), as previously described (Newton et al., 2001, Li et al., 2004, Bauer et al., 2009, Patel et al., 2013). This finding represents an aspect of the well-studied and poorly understood difference between injury-induced regeneration in peripheral and central neurons (Tedeschi, 2011, Cho et al., 2013). Importantly, we found that α_2_δ-1 was also up-regulated in motor neurons following SNL, likely to be a consequence of their axonal damage caused by the ligation (Bauer et al., 2009) (Fig. 6F, G, H).

## Discussion

### Altered expression of hippocampal α_2_δ-1 following kainic acid-induced status epilepticus

Many molecular changes have been described in chronic epileptic tissue. Furthermore, several types of channelopathies were identified in neurons from chronic models of acquired epilepsy. They involve alterations of kinetics of voltage-gated channels, changes in subunit composition, expression of new types of channels or abnormalities of subcellular distribution of specific channels (Poolos and Johnston, 2012). Channelopathies affecting sodium, potassium (Bernard et al., 2004), calcium (Su et al., 2002) or I_h_ (Shah et al., 2004) channels have been described. These alterations represent molecular, structural and functional changes which occur during epileptogenesis and are responsible for increased cellular excitability, altered functional properties of dendrites and conversion from regular firing neurons to bursting neurons. Furthermore, up-regulation of T-type calcium currents was demonstrated in apical dendrites of CA1 neurons following the pilocarpine status epilepticus (Su et al., 2002).

The present results indicate that damage to the central nervous system by kainic acid treatment, which provokes status epilepticus, followed by delayed development of spontaneous seizures in rats (Buckmaster and Dudek, 1997), does not result in a widespread up-regulation of α_2_δ-1 in the hippocampus, at least not at 5 weeks post-kainic acid. Although no overall up-regulation of mossy fiber α_2_δ-1 was observed at the time of this analysis, a reduction of α_2_δ-1 was observed in regions of CA1 pyramidal cell loss. These results may indicate that in the regions of kainate-induced CA1 pyramidal cell loss, the α_2_δ-1 protein associated with presynaptic Ca_V_ channels, in the *en passant* synapses from CA3 neurons or Schaffer collaterals onto CA1 pyramidal neurons, is redistributed once the target CA1 cells and their dendrites are lost (see Figs. 2B and 3A). This may therefore represent a local up-regulation of α_2_δ-1 at neighboring synapses, although to examine this in the future will require quantitative analysis at the electron microscopic level. This increased presynaptic α_2_δ-1 localization could have increased transmitter release as a consequence (Hoppa et al., 2012).

Axonal sprouting of mossy fibers has been found to occur after kainate treatment (McNamara and Routtenberg, 1995). Possible ectopic expression of α_2_δ-1 was observed in the stratum oriens of the CA3 region of some kainate-treated rats that could be associated with mossy fiber sprouting (see Fig. 4Ai), but there was no up-regulation of α_2_δ-1 protein in cell body regions in the dentate gyrus, unlike the situation following peripheral nerve damage.

OX42 up-regulation provides evidence of microglial activation, and has been shown to be an early response to brain injury, including kainate-induced lesions (Akiyama et al., 1994). In the present study we found widespread bilateral microglial activation following kainic acid administration, in all the rats examined that showed status epilepticus, and in one of two rats that did not demonstrate status epilepticus.

### Comparison of histological changes observed in the kainic acid and tetanus toxin-induced seizure models

The hippocampi of rats injected with tetanus toxin only showed localized lesions that mainly coincided with the injection track, and correspondingly localized development of OX42 staining. However, three out of four of these rats developed spontaneous seizures. When compared with the kainate model and other status epilepticus models, the tetanus toxin model possesses distinct features. Tetanus toxin is known to induce chronic epilepsy with spontaneous and recurrent seizures but without morphological changes, and in particular without initial status epilepticus (Jiruska et al., 2013). It was shown previously that hippocampal sclerosis characterized by major cell loss was present in only 10% of animals and affected mainly the CA1 region (Jefferys, 1992, Vreugdenhil et al., 2002, Jiruska et al., 2010, Jiruska et al., 2013). Selective loss of somatostatin-positive interneurons has been demonstrated 8 weeks following tetanus toxin injection (Jefferys et al., 1992). One consistent structural change is axonal sprouting, both in the dentate gyrus (mossy fiber sprouting shown by Timm staining) and in the CA1 region. Based on the results obtained here, we can speculate that changes in α_2_δ-1 distribution observed in the kainate model can be attributed to status epilepticus and/or associated neuronal cell loss.

### α_2_δ-1 up-regulation is a marker of peripheral nerve injury

The α_2_δ-1 subunit is expressed in primary afferent DRG neurons, and is particularly strongly expressed in small DRG neurons, which include C-fiber nociceptors (Bauer et al., 2009). *Cacna2d1*, encoding α_2_δ-1, is one of many genes whose expression is altered following experimental peripheral nerve damage. Indeed, α_2_δ-1 mRNA and protein shows up-regulation following several different types of damage to peripheral axons of DRG neurons, including ligation, chemotherapy and diabetes-induced neuropathy (Luo et al., 2001, Luo et al., 2002, Newton et al., 2001, Davis-Taber and Scott, 2006, Matsumoto et al., 2006, Xiao et al., 2007, Bauer et al., 2009). Furthermore we have found an alteration in α_2_δ-1 splice variant expression in DRGs following peripheral nerve damage (Lana et al., 2013).

The mechanism(s) involved in alteration of gene expression following somatosensory nerve damage are thought to involve propagation of an axonal injury-induced Ca^2+^ wave to the DRG soma, resulting in histone deacetylase 5 export from the nucleus, and activation of gene transcription, a process which has been found to occur in damaged peripheral but not central neurons (Cho et al., 2013).

### Comparison of changes in α_2_δ-1 observed in the models of epilepsy and neuropathic pain

A major difference between the peripheral somatosensory nerve damage that results in α_2_δ-1 up-regulation and kainate-induced neuronal damage is that in the latter there is loss of CNS neurons, in some cases accompanied by sprouting of neighboring neurons, whereas in the former situation, the DRG somata are not killed by the peripheral nerve insult, and the damaged peripheral axons can regenerate. However, although our results do not demonstrate overt or widespread up-regulation of α_2_δ-1 in hippocampal neuronal somata, it is possible that there has been redistribution of α_2_δ-1 at synaptic terminals in areas affected by neuronal cell loss.

### Mechanism of action of gabapentinoid drugs in the alleviation of neuropathic pain

The high affinity binding site for ^3^H-gabapentin in the brain was purified and identified to be α_2_δ-1 (Gee et al., 1996, Brown and Gee, 1998), and it was subsequently found to bind to α_2_δ-2 with similar affinity (Marais et al., 2001, Gong et al., 2001). Autoradiographic studies then identified α_2_δ-1 as the major binding site for ^3^H pregabalin in the rat cerebral cortex, hippocampus and other brain regions, although α_2_δ-2 represented the main binding site in the cerebellum and interpeduncular nucleus (Bian et al., 2006).

We have recently shown that the up-regulation of α_2_δ-1 is required for the rapid development of mechanical hypersensitivity following PSNL, since this is markedly delayed in α_2_δ-1 knockout mice (Patel et al., 2013). It is widely assumed that the up-regulation of α_2_δ-1 in damaged somatosensory neurons is related to the efficacy of the gabapentinoid drugs in alleviating neuropathic pain in humans as well as animal models, since the gabapentinoids are ineffective in mice lacking α_2_δ-1 (Patel et al., 2013), and in knockin mice in which α_2_δ-1 is mutated so that it does not bind gabapentinoid drugs (Field et al., 2006). Nevertheless the mechanism of action of these drugs at the molecular level still remains unclear, as they are generally found to produce little acute inhibition of calcium currents or synaptic transmission (Sutton et al., 2002, Brown and Randall, 2005, Hendrich et al., 2008). However, some studies have found acute effects of these drugs on synaptic transmission (Uchitel et al., 2010).

We have found that the α_2_δ-subunits enhance plasma membrane expression of calcium channels; although the mechanism still remains unclear, it is thought to involve trafficking of the channels from their site of synthesis to the plasma membrane (Canti et al., 2005, Bauer et al., 2010). It is likely that in neurons α_2_δ subunits have multiple effects on calcium channel distribution, both associated with long-range calcium channel trafficking from their site of synthesis in the soma to their mainly presynaptic localization in nerve terminals (Bauer et al., 2009), and also local effects on calcium channel localization in membrane micro-domains such as the active zone and in lipid rafts (Davies et al., 2006, Hoppa et al., 2012), as well as influencing the recycling of calcium channels to the plasma membrane (Tran-Van-Minh and Dolphin, 2010).

Furthermore, we have found that the gabapentinoid drugs have an inhibitory effect on calcium currents when applied over longer time periods, in cultured cells and neurons (Hendrich et al., 2008, Tran-Van-Minh and Dolphin, 2010), which we infer is by inhibiting the trafficking of the α_2_δ subunits (Hendrich et al., 2008, Bauer et al., 2010, Tran-Van-Minh and Dolphin, 2010). We also observed *in vivo* that there was less up-regulation of α_2_δ-1 in nerve terminal zones, after the induction of somatosensory nerve injury when it was combined with chronic pregabalin treatment (Bauer et al., 2009), which might be an effect on long range axonal trafficking, or on lifetime of the protein and its local recycling at presynaptic terminals. Our evidence currently indicates that this interference by gabapentinoids with the function of α_2_δ-1 and α_2_δ-2 results in a reduction of expression of the entire calcium channel complex at the plasma membrane (Tran-Van-Minh and Dolphin, 2010, Cassidy and Dolphin, 2014). In agreement with this, we have also observed inhibitory effects of chronically applied gabapentinoids on excitatory synaptic transmission from DRG neuron terminals (Hendrich et al., 2012).

### Role of α_2_δ subunits and mechanism of action of gabapentinoids in epilepsy

The gabapentinoid drugs have therapeutic efficacy as antiepileptic drugs, although they are generally used in combination therapy (Marson et al., 2000, Glauser et al., 2006). In animal models of seizures, gabapentin has been shown to be effective (Cilio et al., 2001). However, the mechanism of action of the gabapentinoid drugs in epilepsy is poorly understood. Although both gabapentin and pregabalin were first developed to enhance GABA-ergic inhibition in the brain (Taylor et al., 1992, Taylor et al., 2007, Silverman, 2008), it is now clear that they do not act by mechanisms involving inhibition of GABA breakdown, or activation of GABA-A or GABA-B receptors (Taylor et al., 2007, Li et al., 2011). Furthermore GABA itself does not bind to the α_2_δ subunits that are now known to be the target for gabapentinoid drugs (Li et al., 2011).

Although it has been found that α_2_δ-1 is the target for the gabapentinoid drugs in the alleviation of experimental neuropathic pain in rodents, (Field et al., 2006, Patel et al., 2013), this is not known for the efficacy of the gabapentinoids in animal models of epilepsy. An *in situ* hybridization study showed that α_2_δ-1 expression was often more associated with excitatory neurons, and α_2_δ-2 with inhibitory neurons (Cole et al., 2005). The α_2_δ-1 protein is strongly expressed in the hippocampus, therefore it is possible that a change in expression of α_2_δ-1 in epileptic foci might influence the effectiveness of the gabapentinoid drugs. In contrast α_2_δ-2 is expressed in a more restricted pattern, for example it is strongly expressed in cerebellar Purkinje neurons (Barclay et al., 2001, Brodbeck et al., 2002). The loss of expression of α_2_δ-2 in *cacna2d2* mutant mouse strains including “Ducky” results in cerebellar ataxia and spike-wave epilepsy, and is associated with severe Purkinje cell dysfunction (Barclay et al., 2001, Brodbeck et al., 2002, Brill et al., 2004, Donato et al., 2006). Furthermore *CACNA2D2* is disrupted in rare recessive human cases of epileptic encephalopathy (Edvardson et al., 2013, Pippucci et al., 2013). Therefore interference with α_2_δ-2 function might be intuitively less likely to be the therapeutic target of the gabapentinoids in epilepsy, compared to disruption of α_2_δ-1 function. However, this would not exclude the possibility that there is localized alteration of α_2_δ-2 expression, which could be a therapeutic target in focal (partial) epilepsy.

Thus it is possible that α_2_δ-2 levels or distribution might be affected in animal models of epilepsy. Unfortunately there are currently no available α_2_δ-2 antibodies that are effective in immunohistochemistry, so at present this cannot be easily tested. However, it would be extremely useful to examine whether gabapentin is effective in epilepsy models, using knockin mice in which either α_2_δ-1 (Field et al., 2006) or α_2_δ-2 is mutated so that it is gabapentin-insensitive (Lotarski et al., 2011).

### Non-calcium channel functions of α_2_δ proteins

Recently, α_2_δ-1 has been found to interact with thrombospondins (Eroglu et al., 2009), and this interaction has been shown to be involved in synaptogenesis, a process which has been described as being independent of its function as a calcium channel subunit. Thrombospondins are a ubiquitous family of extracellular matrix proteins, which are secreted by many cell types, including microglia (Chamak et al., 1995, Eroglu et al., 2009). In a number of experimental and human epilepsies there is reactive gliosis (Seifert et al., 2010), microglial activation (Avignone et al., 2008), and axonal sprouting (Tauck and Nadler, 1985, McNamara and Routtenberg, 1995, Vreugdenhil et al., 2002, Sutula and Dudek, 2007). It has also been proposed that gabapentin inhibits the interaction between α_2_δ-1 and thrombospondins, and therefore inhibits synaptogenesis (Eroglu et al., 2009). Although this might be considered a plausible mechanism of action of gabapentinoid drugs in treatment of epilepsies, synaptic remodeling that occurs at epileptic foci (Lew and Buckmaster, 2011) is likely to have already occurred before the onset of treatment with these drugs. Nevertheless, it is possible that the gabapentinoid drugs may also modify epileptogenesis and decrease the consequences of status epilepticus by reducing cellular damage and seizure frequency (Cilio et al., 2001, Li et al., 2012).

## Author contributions

A.C.D. and J.G.R.J. conceived the study. P.J. performed *in vivo* procedures and monitoring. M.N.-R. performed and analyzed all hippocampal histology with the help of G.S. C.S.B. and M.N.-R. performed all DRG and spinal cord histology. A.C.D. and M.N.-R. wrote the paper, with input from all authors.
